# Approach–Avoidance Bias in Virtual and Real-World Simulations: Insights from a Systematic Review of Experimental Setups

**DOI:** 10.3390/brainsci15020103

**Published:** 2025-01-22

**Authors:** Aitana Grasso-Cladera, John Madrid-Carvajal, Sven Walter, Peter König

**Affiliations:** 1 Institute of Cognitive Sciences, Osnabrück University, 49090 Osnabrück, Germany; aitana.grasso.cladera@uni-osnabrueck.de (A.G.-C.); john.madridcarvajal@uos.de (J.M.-C.); sven.walter@uni-osnabrueck.de (S.W.); 2 Department of Neurophysiology and Pathophysiology, Center of Experimental Medicine, University Medical Center Hamburg-Eppendorf, 20251 Hamburg, Germany

**Keywords:** approach–avoidance bias, virtual reality, ecological validity, systematic review, embodied cognition

## Abstract

Background: Approach and avoidance bias (AAB) describes automatic behavioral tendencies to react toward environmental stimuli regarding their emotional valence. Traditional setups have provided evidence but often lack ecological validity. The study of the AAB in naturalistic contexts has recently increased, revealing significant methodological challenges. This systematic review evaluates the use of virtual reality (VR) and real-world setups to study the AAB, summarizing methodological innovations and challenges. Methods: We systematically reviewed peer-reviewed articles employing VR and real-world setups to investigate the AAB. We analyzed experimental designs, stimuli, response metrics, and technical aspects to assess their alignment with research objectives and identify limitations. Results: This review included 14 studies revealing diverse methodologies, stimulus types, and novel behavioral responses, highlighting significant variability in design strategies and methodological coherence. Several studies used traditional reaction time measures yet varied in their application of VR technology and participant interaction paradigms. Some studies showed discrepancies between simulated and natural bodily actions, while others showcased more integrated approaches that preserved their integrity. Only a minority of studies included control conditions or acquired (neuro)physiological data. Conclusions: VR offers a potential ecological setup for studying the AAB, enabling dynamic and immersive interactions. Our results underscore the importance of establishing a coherent framework for investigating the AAB tendencies using VR. Addressing the foundational challenges of developing baseline principles that guide VR-based designs to study the AAB within naturalistic contexts is essential for advancing the AAB research and application. This will ultimately contribute to more reliable and reproducible experimental paradigms and develop effective interventions that help individuals recognize and change their biases, fostering more balanced behaviors.

## 1. Introduction

Approach–avoidance behaviors are automatic responses organisms exhibit toward environmental stimuli, shaped by the positive or negative value of those stimuli [1,2]. Organisms continuously interact with their surroundings, assessing objects, events, and opportunities based on perceived benefits or threats. The tendency to approach or avoid stimuli is modulated by their valence or emotional value: organisms are quicker to approach stimuli perceived as rewarding or beneficial for safety and well-being [3,4,5]. Conversely, organisms tend to avoid stimuli linked to adverse or harmful outcomes, aiming to protect themselves from threats that could compromise their safety [3,6]. Emotional valence is central to regulating approach–avoidance behaviors, with responses driven by an underlying positive–negative valence behavioral system that influences interactions in meaningful ways [1,7,8].

### Approach–Avoidance Bias

Empirical evidence shows that approach–avoidance behaviors are influenced by the congruency of action and the emotional valence of the stimulus and may involve an embodied component. Studies show that organisms respond faster and more accurately to stimuli aligned with their instincts—approaching positive stimuli and avoiding negative ones—than to stimuli that conflict with these predispositions [9,10,11,12,13,14]. This automatic approach–avoidance bias (AAB) suggests an inherited tendency to perform actions automatically, guided by perceptual, experiential, or cognitive biases [6]. Recent research highlights an inherent embodied component of the AAB, rooted in its evolutionary role, emphasizing the need to study it in dynamic, realistic contexts [6,15]. The embodied aspect of the AAB suggests that physiological mechanisms regulate this automatic modulation of actions, offering insights into how bodily dynamics shape decision-making processes and responses to environmental stimuli.

Research on the AAB encompasses diverse methodological approaches, employing various stimuli, tasks, and measures to address a wide range of questions. A key method is the stimulus–response compatibility (SRC) task, particularly the widely used approach–avoidance task (AAT), where participants perform an action symbolizing a specific movement (e.g., pushing or pulling via button pressing) or actually performing the actions using, for example, a joystick, in response to emotionally valenced stimuli while their reaction times are measured. Standard behavioral metrics to measure an AAB include response time (i.e., time taken to perform a specified action) and accuracy (i.e., alignment of action with instruction) [5,11]. Additional measures, such as ocular dynamics (e.g., saccades, fixations) for attentional processes [16,17] and autonomic nervous system activity —cardiac activity [18] and skin conductance [19]—have provided insights into vagal tone, stress responses, and emotion regulation [20,21,22]. This diverse set of techniques and metrics enriches our understanding of the AAB by enabling the study of its behavioral and physiological underpinnings across varied contexts.

The AAT has been a popular paradigm for studying AAB due to its versatility in addressing various research topics. Nevertheless, the AAT and its variations have primarily been implemented as stationary, computer-based tasks that are highly controlled and tend to present non-realistic scenarios with fewer degrees of ecological validity, i.e., low representativity of stimuli and relevance of tasks within the context of functional action [23]. In this sense, real-world paradigms can offer newer insights on embodied aspects of the AAB (i.e., different bodily actions for approaching or avoiding and physiological dynamics), which are fundamental to further understanding, for example, motivation processes and the evolutionary role of the bias [9]. Overall, virtual reality (VR) and real-life setups offer researchers the opportunity to deviate from classical paradigms by creating realistic scenarios to study the AAB under controlled yet ecologically valid conditions.

The VR setup allows researchers to collect detailed behavioral data, including head, eye, hand, and body movements during participant interactions with virtual objects, offering insights beyond reaction time and response accuracy into the embodiment of AAB [5,11]. Also, VR devices open up the possibility of being combined with other measurement devices, facilitating the acquisition of multimodal data, such as from physiology, e.g., electrical brain and cardiac activity [24,25,26]. Implementing experimental paradigms based on VR opens the door for new research questions, allowing for a paradigm shift to further understand the behavioral and embodied mechanisms involved in the AAB by performing naturalistic actions in a scenario closer to real life.

Previous research has demonstrated the feasibility of bringing the AAB study into VR. For example, Degner and collaborators [1] assessed the AAB in an immersive virtual environment, where they could replicate the main findings reported for desktop-based paradigms for the AAB. Also, the use of VR in the context of the AAB has been reported for the development of innovative treatments for different conditions (e.g., nicotine addiction and eating disorders). An example of this is the line of work conducted by Eiler and collaborators [4,27,28], who have systematically implemented an AAB paradigm in VR to generate a cognitive bias modification (CBM, i.e., an approach to modify automatic cognitive processes in a specific direction [29]) paradigm for the treatment of smoke/nicotine addiction [30]. Hence, VR’s integration into AAB research confirms its feasibility for both replicating traditional findings and advancing therapeutic applications. These developments underscore VR’s potential as a valuable tool for treatments.

However, implementing these paradigms in VR is non-trivial and requires careful assessment of methodologies and the resolution of several challenges. For instance, a preliminary evaluation of transferring the AAB research into VR focusing on improved usability, functionalities, and graphics showed that Leap Motion sensors (i.e., a visual body-tracking module that precisely captures hand and finger movements) are preferred to controllers [4]. This transfer brings challenges such as the accuracy of time recording, the gesture or action to be performed and recorded, as well as rethinking the inclusion of the analysis of eye, head, hand, and body movements in light of the AAB. Hence, issues such as timing accuracy, choice of gestures, and using sensors like Leap Motion over controllers highlight the need for advanced usability, precise tracking, and comprehensive analysis of movements to fully adapt AAB studies to virtual settings.

This systematic review provides a comprehensive overview of the current literature that uses VR or real-life setups to study the AAB. Specifically, we focused on (1) examining and summarizing methodological aspects of the existing literature. This thorough examination of the methods employed provides insights into how studies have approached using VR and real-life setups to study the AAB. Also, we aimed to (2) highlight the major limitations and challenges associated with using VR to study the AAB. Overall, with this systematic review, we attempted to establish a path for future research studying the AAB in setups with higher ecological validity for a proper understanding of the mechanisms underlying the AAB and its embodied dynamics.

## 2. Methods

The present review was conducted according to the Joanna Briggs Institute (JBI) guidelines for systematic reviews and meta-analyses [31] and is reported according to the Preferred Reporting Items for Systematic Reviews and Meta-analyses (PRISMA) [32,33]. It was not registered; a protocol can be found in the Open Science Framework (OSF) (the protocol can be found in the following link: https://osf.io/huv6x/).

### 2.1. Eligibility Criteria

This review focuses on studies conducted either in VR environments or in natural settings with a high degree of ecological validity to explore the phenomenon of the AAB. Given the diverse range of experimental designs in neuroscience and cognitive science research, no specific inclusion or exclusion criteria were applied based on the study design. As a result, all experimental and quantitative study designs published in peer-reviewed journals were included. However, due to the focus on methodology and analysis, study protocols were excluded. In terms of sample characteristics, only studies involving human populations were considered. This includes research on both healthy individuals and specific target groups (e.g., those with clinical conditions).

Since this review emphasizes the methodological and analytical aspects of the AAB, studies using an approach–avoidance task in VR or natural settings as a training or treatment tool for specific conditions were excluded. In this sense, we only included articles that aimed to observe, explore, and evaluate the AAB unrestrictedly. All other experimental paradigms aimed at studying the AAB in these settings were eligible for inclusion. Additionally, there were no restrictions related to comparison conditions, meaning the review includes studies with or without control conditions.

There were no time restrictions, and only studies published in English were considered. Table 1 provides a summary of the study selection criteria.

### 2.2. Information Sources

The searches were conducted using Web of Science (WoS; 1945 onwards), PUBMED (1948 onwards), and Scopus (1969 onwards). Additionally, literature search tools, such as Research Rabbit [34] and Connected Papers [35], were utilized to enhance the likelihood of identifying all relevant published articles on the topic. Reference lists of the included studies and any relevant reviews were also thoroughly scanned for further sources. Searches were conducted during October 2024.

### 2.3. Search Strategy

Search strategies for systematic reviews were developed using keywords related to approach–avoidance bias, virtual reality, and naturalistic setups. The final search strings were refined by reviewing the keywords used in various articles within the field, alongside synonym keywords for approach–avoidance bias, virtual reality, and natural or real-world setups. Supplementary Material File S1 provides the specific search terms and an example of the search strategy for one database.

### 2.4. Study Records

#### 2.4.1. Data Management

The literature search results were downloaded from each database and consolidated into an Excel document (Microsoft Corporation, Redmond, WA, USA) to facilitate collaboration among reviewers during the selection and data extraction phases. After removing duplicates, the refined database was uploaded to ASReview software (v1.0) [36] for screening. A calibration exercise was first conducted to train and refine the screening process before it began. Reviewers were then trained to use the ASReview software before starting the screening.

For data extraction, the authors created a Google Form to gather relevant information from the selected articles. A similar calibration and training procedure was performed before the data extraction phase began.

#### 2.4.2. Selection Process

The selection process was carried out by three independent reviewers (IRs), under the supervision of two authors (AGC and JMC). Initially, the IRs jointly screened several articles as a calibration exercise. Once a high level of consistency was reached (95%), the IRs proceeded to independently screen the remaining titles and abstracts found in the search, using the inclusion criteria within the ASReview software. The software was trained to maximize the benefits of its machine-learning algorithm. Complete reports were obtained for all titles that met the inclusion criteria (Table 1) or where uncertainty remained. The IRs then reviewed the full text of these articles to determine whether they should be included. Any disagreements were resolved through discussions between the IRs and the principal review authors. All reasons for excluding articles were carefully documented. Both the review authors and the IRs had access to the journal titles, study authors, and institutions involved.

#### 2.4.3. Data Collection Process

The review authors (AGC, JMC, and PK) created a Google Form questionnaire and a detailed instruction manual to guide the extraction of relevant information from each included article. Following a training and calibration phase, three IRs performed the data extraction under the supervision of AGC and JMC. The Google Form contained specific questions designed to address various aspects of the research question. Any disagreements during the extraction process were resolved through discussions between the IRs and the authors.

### 2.5. Data Items

In this review, various types of information were extracted from all included articles. These included bibliographic details such as author names, journal titles, year of publication, and DOI. We also collected specific information about the study content, including the main objectives and the nature of the research. Additionally, paradigm-related details, such as the type of task and stimuli presented, were recorded. Given the focus of this review, methodological information was also gathered, including the devices employed, the type of action performed, and behavioral data recorded. Finally, we extracted details about the reported limitations and suggestions for future research on using VR to study the AAB. A comprehensive list of data items can be found in Supplementary Material File S2.

### 2.6. Risk of Bias

JBI Critical Appraisal Tools [37,38] were used to assess the potential risk of bias for each study. For each article, the tool’s corresponding extension regarding the experimental design was employed. Two authors (AGC and JMC) conducted this step.

### 2.7. Data Synthesis

This review contains text, tables, and figures to summarize and explain the primary characteristics and findings of the included studies. The narrative synthesis method allowed us to establish relationships and findings between the included studies [39]. Due to significant heterogeneity among the included studies—in their aims, designs, and methodological characteristics—as well as inconsistencies in measured outcomes, a meta-analysis was not conducted [40,41]. The lack of uniformity across studies would make establishing reliable comparators challenging and could lead to potentially misleading generalizations. Consequently, a narrative synthesis was favored to better capture the nuanced insights each study provided.

## 3. Results

### 3.1. Study Selection

**Figure 1:**
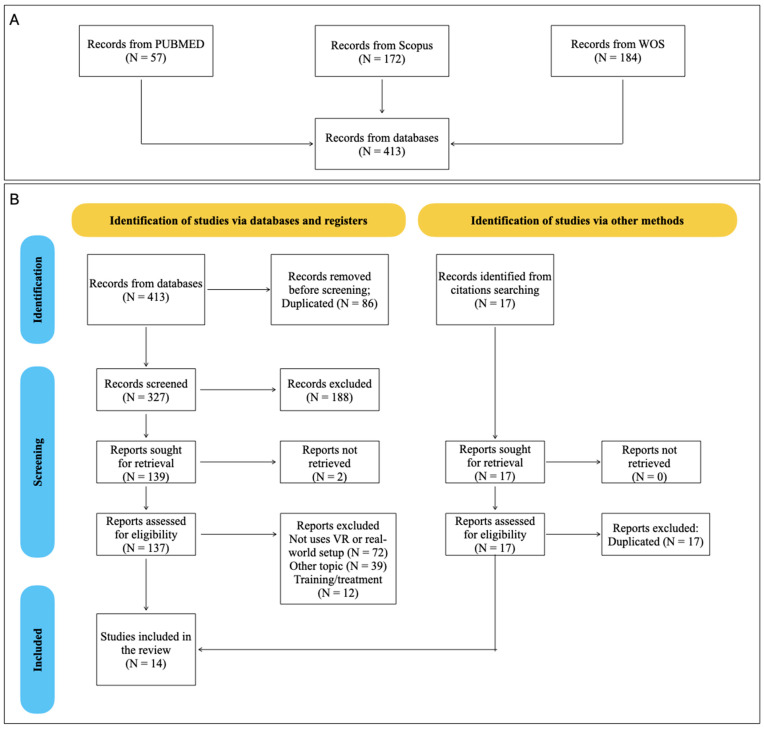
Flow of articles through the different stages of the review. Panel (**A**) shows the number of articles found per database. Panel (**B**) presents the PRISMA flowchart.

Following the initial search (see Supplementary Material File S1), 413 articles were found to be potentially eligible. Figure 1 illustrates the number of considered articles through the different stages. The initial number of articles was reduced to 327 after removing 86 duplicates. To select the articles, we conducted two screening processes. The first screening was conducted based on the information presented in the article’s title, and 188 articles were excluded. We searched the full text of the remaining articles, and only two were not accessible. Then, we conducted the second screening, grounded on the information provided in the articles’ abstract and full texts when required; 137 articles were excluded. The search conducted in other browsers gave results that duplicated the articles found in the other databases; hence, they were not included. Another 7 articles were excluded during the data extraction process since they did not fulfill one or more of the specific criteria for this review.

One of the main reasons for excluding articles was the absence of the use of VR or natural/real-world setup to study the bias (N = 72). Secondly, many articles (N = 39) were excluded because they were unrelated to the AAB or because they aimed at studying the automatic implicit bias (AIB). The AAB refers to the instinctive tendency to move toward positive stimuli and away from negative ones, often guided by perceived emotional valence, like pleasure or threat. In contrast, AIB involves unconscious associations or stereotypes toward groups or categories (e.g., race, gender) that shape perception and behavior without conscious awareness [42,43,44]. While the AAB arises in the immediate physical or emotional responses to stimuli, the implicit bias operates through ingrained attitudes that subtly influence decisions and judgments. Finally, it is possible to see many studies using VR (N = 44) that were excluded in both screening processes, since they used the VR environment as a training or treatment. However, this shows the interest in moving towards more realistic setups. This procedure resulted in a final sample of 14 articles.

### 3.2. Studies Characteristics

**Figure 2:**
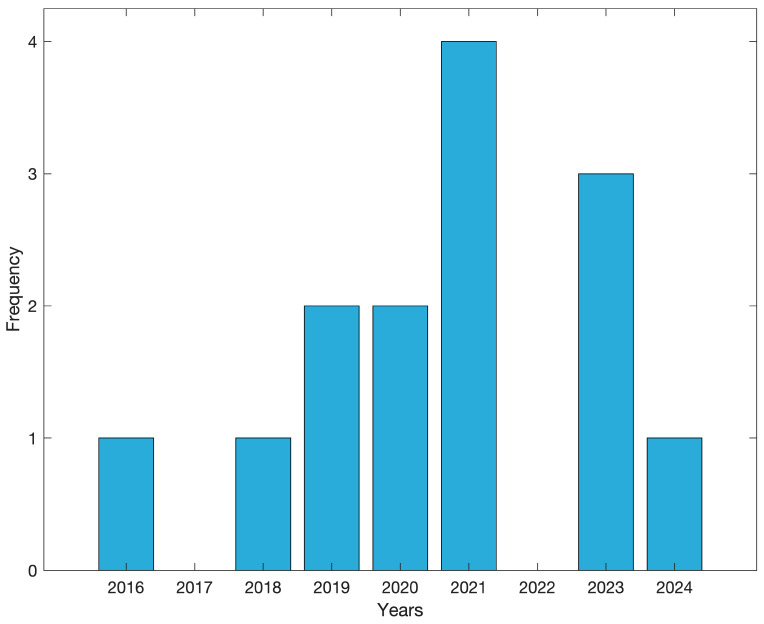
Number of included articles per year.

The studies included in this review were published between 2016 and 2024. Our results show that an increasing number of articles in the last three years have used VR or a real-world setup to study the AAB (Figure 2).

The included articles are diverse. This is evident since the research question and the main objective of the included studies differ from one to the other. For example, some articles explore a behavioral bias towards food items [45,46,47,48], while others explore some domains of social interaction [49,50,51,52]. On the other hand, two of the included studies explored differences regarding psychiatric disorders or personality traits. Also, some of the included articles aimed to explore how approach–avoidance behaviors are modulated when using a VR environment [1,4]. Other studies explored fear [53,54], gaming disorder [55], and alcohol intake [56]. The diversity of the research question and objective of the included studies highlights the versatility of the AAB framework to be applied to different research topics.

The diversity regarding the research question is also translated into a variation, such as the articles included. In this sense, some of the included articles aimed to explore behavioral aspects of the AAB linked to different conditions (e.g., social interactions and food preference, among others), while other articles aimed to prove the feasibility of studying the AAB in VR. However, some articles addressed both behavioral and feasibility aspects simultaneously. These articles, respectively, can be categorized as Empirical [1,4,45,46,47,48,49,50,51,52,53,54,55,56] and Methodological [1,4,47,50,52,55,56] regarding their primary objective. All the included articles had a question or objective aiming to explore the bias in VR. At the same time, only half of them were also aimed at testing methodological aspects of moving the study of the AAB from a desktop-based setup to VR.

Interestingly, all the articles included examined the AAB within a VR setup, and no studies investigating the AAB in real-world settings were included in the analysis.

### 3.3. Risk of Bias Results

Table 2 summarizes the risk of bias evaluation outcomes, showing that most included articles meet the tool’s criteria for medium to high quality. Although minor details were occasionally missing from the experimental setup descriptions, this did not impact the studies’ clarity or reproducibility. Additionally, only studies that conducted control measurements of the VR setup (e.g., desktop-based or real-life comparisons) or included comparisons across different population groups were categorized as case–control studies.

### 3.4. Paradigm Characteristics

We reviewed paradigm-related experimental characteristics from each one of the included studies. These characteristics involve the type of task (Due to the large differences between tasks, a systematization is not possible. Hence, we have opted for presenting the description of each task in detail in the extended results at https://osf.io/huv6x/) used to study the bias, the stimuli the participants had to interact with, the bodily response given by the participants, the type of behavioral data collected, and the presence of a comparison condition (in methodological terms). By systematizing these characteristics, we can provide insights into the different approaches used to study the bias in VR.

Regarding the experimental approach of the included articles, most (N = 7) implement a version of the classical AAT used to study the bias [1,45,48,50,51,52,55,56]. Several of the included studies had an interactional or distance reduction task regarding virtual agents [49,51,52]. Two of the included experiments were task-destinated to the exploration of either a cave or a maze [53,54]. Finally, one study implemented a sorting task [4], and another one implemented a decision-making task [46]. Table 3 presents a brief summary of each experimental paradigm implemented.

We explored the number of trials conducted in each one of the experiments of the included studies. On average, the included tasks had 136.8 trials (std = 102.08; min = 20; max = 400). Interestingly, four of the included articles had tasks with less than 50 trials [4,50,51,52], only one had between 51 and 100 [55], and another one had between 100 and 150 trials [1]. Five experiments asked the participants to complete between 151 and 200 trials [46,48,51,52,56], and only one had 400 trials [47]. In three cases [49,53,54], it was not possible to determine how many trials were performed by the participants. These results indicate a clear preference for designed experiments with increased trial numbers, enhancing key analytical aspects such as statistical power.

We found a substantial diversity of stimuli used to measure the AAB across all included articles. Most of the included articles implemented 3D stimuli in their experimental designs, and only one used 2D images within the VR environment. Regardless of the actual content of the stimuli or prime used by each study, which is highly related to its research question, 13 of the 14 included studies used 3D stimuli displayed in the VR environment [1,4,45,46,47,48,49,50,51,52,53,54,55]. A variation similar to the research question was found in terms of the content since each article incorporated different stimuli considering its primary objective. Examples of the used stimuli are 2D images of alcoholic vs. non-alcoholic beverages, 3D virtual representations of food vs. non-food items, smoke vs. non-smoke items, and virtual avatars. Across studies, stimuli served different contextual purposes, such as mimicking real-world social interactions, ecological tasks, and fears by allowing participants to interact with virtual objects, agents, and environments. Despite this diverse implementation of stimuli, an observed commonality concerns the common goal of evoking context-specific automatic responses relevant to approach–avoidance tendencies.

**Figure 3:**
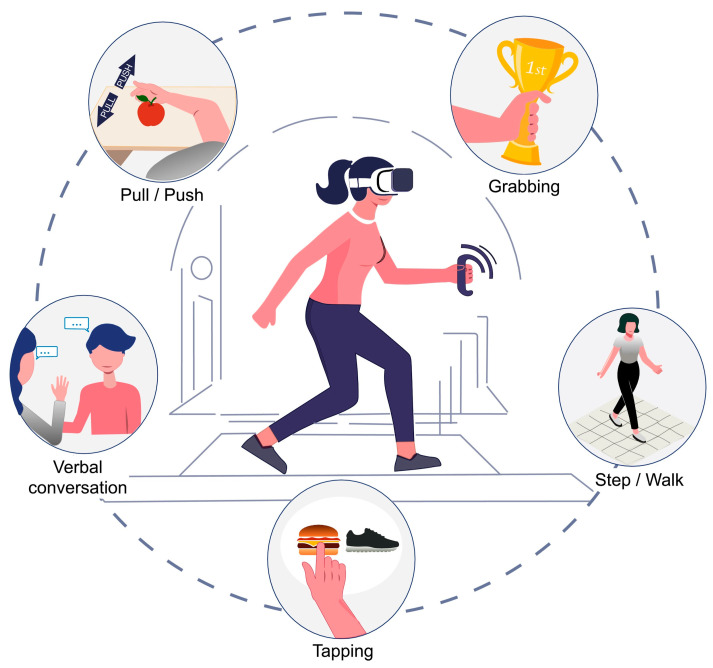
Graphical representation of embodied movements used in VR to study the AAB. The central panel of the image shows a subject immersed in a VR environment. The five actions around the circle display the different embodied movements used in the included studies to measure participants’ responses.

Also, we found that the tendency to include bodily movements as responses is present in all the included articles. All the included articles employed some level of embodied movement in the responses from the participants. From pushing/pulling movements [4,51,55,56] using, for example, a joystick, which is one of the most common types of movements used when studying the AAB, or a hand gesture, to stepping or freely walking [1,50,51,52,53,54], grabbing [45,46,47,56], and even tapping [48] and engaging in verbal conversations [49] (Figure 3). These results show the multiplicity of actions that can be explored beyond pushing and pulling when it comes to studying the embodied mechanisms of the AAB, which encourages thinking about new directions to consider, especially when trying to study the AAB as it occurs outside the laboratory. Similarly, it highlights the versatility of using a VR environment when studying embodied dynamics since the possibilities of movements are more extensive than those implemented in desktop-based experiments.

Regarding the behavioral data collected, the reaction time is one of the primary measures for the AAB. Coherent with previous research in desktop-based computers, the reaction time was one of the most common behavioral measures used by the VR studies [1,4,45,46,47,48,51,52,55,56]. It is important to notice that some experiments recorded more than one behavioral measure, so they performed multiple analyses. The type of action performed by the participants was also a standard measure [49,50,52,53], followed by the position or distance of the participants in the VR environment [1,47,51,54], the exploration time [53,54], and the velocity [52]. This exemplifies that, even when the bias has been studied mainly as a reaction-time-based task, different behavioral components can shed light on the understanding of the dynamics of the bias.

Finally, we found that most of the included studies did not incorporate a control task in terms of setup. Of the total reviewed studies, only four conducted a control experiment where the bias was explored in a desktop-based AAT condition to have a control measure or determine differences with the data collected from VR. Three out of the four studies with a control condition used the same stimuli presented in VR for the desktop condition (most of the time, using screenshots of the same objects). This allowed the evaluation of the stimuli as a valid prime to elucidate the bias.

Table 4 provides details about the paradigm characteristics described before.

### 3.5. Technical Characteristics

Due to the methodological emphasis of the present review, we included the systematization of characteristics related to technical aspects of the experimental setups. We extracted characteristics from the used VR headsets (e.g., brand, sampling rate), controller type, participants’ position and possibility of movement during the experiment, and the system to generate or display the environment. We assessed if any type of (neuro)physiological data were acquired during the experiment (e.g., brain or cardiac activity, skin conductance, among others). The information provided here is based on what is reported in each article; no additional information was included through research. The systematization of these characteristics might promote and guide the development of new experimental setups. A detailed description of these categories can be found in Supplementary Material File S2.

Regarding the type of VR device used for displaying the environment, head-mounted displays (HMDs) were the most common. Only two of the included studies implemented glasses instead of classical HMD VR equipment [51,54]. Brands and manufacturers varied across the studies, *Vive* being the most common one [4,47,50,51,52,55,56]. The resolution of the environment and the sampling rate for collecting behavioral data (e.g., position) were also diverse for the different studies. However, seven of the included articles did not report resolution information [45,47,48,49,50,53,55], and eight did not offer any insights regarding the sampling rate [45,46,49,50,53,55,56]. Most of the controllers were apparatuses from the same brand as the HMD. Six of the systems used for creating and displaying the virtual environment implemented some version of Unity [1,4,45,46,47,53], while two used Vizard [51,52]. There was no information regarding this parameter in four of the included studies [48,50,55,56].

Only two of the included studies performed measures concerning the collection of (neuro)physiological activity measuring brain activity [46,53]. One study implemented fNIRS [46], while the other used EEG and ECG [53].

Finally, regarding the physical positioning of the participants during the experiment, it was more common for them to be sitting down and performing a hand–arm movement [45,46,47,48,55,56] or standing while stepping or walking [4,50,51,52,53,54]. In one study, participants performed pulling and pushing movements despite using an experimental design involving a standing position [51]. Similarly, one study required participants to be seated but engage in a verbal conversation [49]. Only one study incorporated both leaning movements (e., forward and backward body inclinations) while participants were seated and stepping movements (e.g., forward and backward steps) while participants maintained a standing position [50].

Table 5 provides details about the technical characteristics described before.

### 3.6. Limitations and Future Direction

This review analyzes studies using VR or real-world paradigms to examine the AAB. To keep this focus, we report only the limitations and future directions from the included articles that directly pertain to the implementation of VR or real-world setups. This review does not address broader limitations, such as sample size, participant characteristics, or suggestions like investigating setup feasibility as a treatment or its application in other populations. These aspects fall outside the review’s primary focus, which is to provide a comprehensive overview of current literature using VR or real-life setups to study the AAB. Consequently, such suggestions are beyond the scope of this analysis. Accordingly, Table 6 presents limitations and future directions from the articles that specifically impact the application of VR or real-world setups for studying the AAB and might be helpful for the development of future research.

## 4. Discussion

This systematic review provides a detailed overview of the current literature utilizing VR or real-life paradigms to investigate the AAB. By examining the diverse paradigm and methodological characteristics, we offer a narrative synthesis of evidence that supports the feasibility of implementing the AAB in more flexible, ecologically valid environments while preserving its behavioral properties. The synthesized information reveals the significant challenges of transferring the AAB from traditional paradigms, highlighting a wide range of stimuli, embodied responses, and collected behavioral data. The studies included in this review varied regarding virtual environments, devices, resolution, and sampling quality. In the following section, we will discuss additional factors observed during this review and the implications of our findings for the AAB research. We will explore the methodological considerations reported for translating traditional paradigms into virtual environments and examine the impact of diverse stimuli and response measures on research outcomes. We will also highlight potential future research directions, emphasizing the importance of methodological consistency, ecological validity, and additional behavioral measurements in understanding the AAB within immersive settings.

### 4.1. How Homogeneous Is the Study of the AAB Using VR?

While the primary aim of this systematic review was to examine how the study of the AAB has been implemented using VR and real-world settings, a substantial portion of the literature diverges toward treatment and training applications. These studies often suggest that the AAB can be effectively transferred into VR without delving into its underlying behavioral components or the suitability of the measurements collected. Examples include Jahn et al. [57] and Smeijers et al. [58], which focus on health-related outcomes and aggressive impulse management. Additionally, many studies explore automatic implicit biases, which often are modulated by other factors like moral judgment rather than directly examining the AAB. For instance, studies by Banakou et al. [59], Kishore et al. [60], Peck et al. [61], You et al. [62], and Persky et al. [63] investigate racial bias through virtual embodiment techniques. Echoing this line of inquiry, Banakou et al. (2020) examined how social context affects the feeling of owning one’s body and contributes to implicit racial bias. This orientation reflects a broader trend, where VR research is primarily used to address not only socio-psychological biases related to ethnicity but also physical disability [64] and social anxiety [65]. While such applications are valuable, they fall outside the immediate scope of understanding the AAB’s behavioral nuances as they occur in real-world dynamic situations. By assuming the methodological and behavioral elements, movements, and technologies involved, these studies neglect the crucial need for a foundational common framework that would eventually allow for accurate translation of the AAB research into VR. Without this groundwork, the legitimacy of results and their application to clinical populations remains questionable, as the essential task of establishing baseline principles for VR integration into the AAB research has not been adequately addressed. Together, these divergent studies highlight a gap in the literature where future research should aim to disentangle the behavioral elements that characterize the AAB from broader bias investigations related to it. This approach would ensure that findings directly affect the theoretical understanding and practical deployment of the AAB in VR settings.

### 4.2. From Healthy Subjects to Clinical Populations

Besides measuring cognitive biases, improving methods (or developing novel ones) for intervention and treatment of AAB-related biases is crucial for counteracting their negative impact (e.g., substance abuse) on individuals and groups. Effective interventions could help individuals recognize and modify their biases, fostering more balanced behaviors. Some of the studies included in this review investigated AAB tendencies using VR within clinical populations for therapeutic purposes. These studies focused on diverse conditions, such as psychopathy [51], alcohol abuse [56], internet gaming disorders [55], and habitual food avoidance in anorexia [48], highlighting variations in inhibitory control mechanisms and demonstrating the feasibility of conducting AAB research using VR setups that extend beyond traditional methods like button presses and joystick manipulation. Furthermore, it is important to consider that the AAB in laboratory settings is not exclusively generated by the presentation of a single picture or stimulus but is the result of the underlying explicit task. This means that the behavioral response generated by the agents is modulated by the context and the explicit task they are presented with, where the valence of the stimuli has a moderator role on the task’s performance [66,67,68]. However, our understanding of the bias itself within real and virtual contexts remains limited due to the recent emergence of such studies, which vary significantly in their methodologies and aims. This diversity highlights a gap in the literature, underscoring the need for further research to comprehensively examine the AAB within these environments to better grasp its fundamental characteristics and applications. Further research in this area can contribute to establishing flexible therapeutic interventions and developing preventative strategies that target bias formation and maintenance.

### 4.3. Mapping the Terrain: An Examination of the Relationships Between Characteristics of the AAB Research Using VR

**Figure 4:**
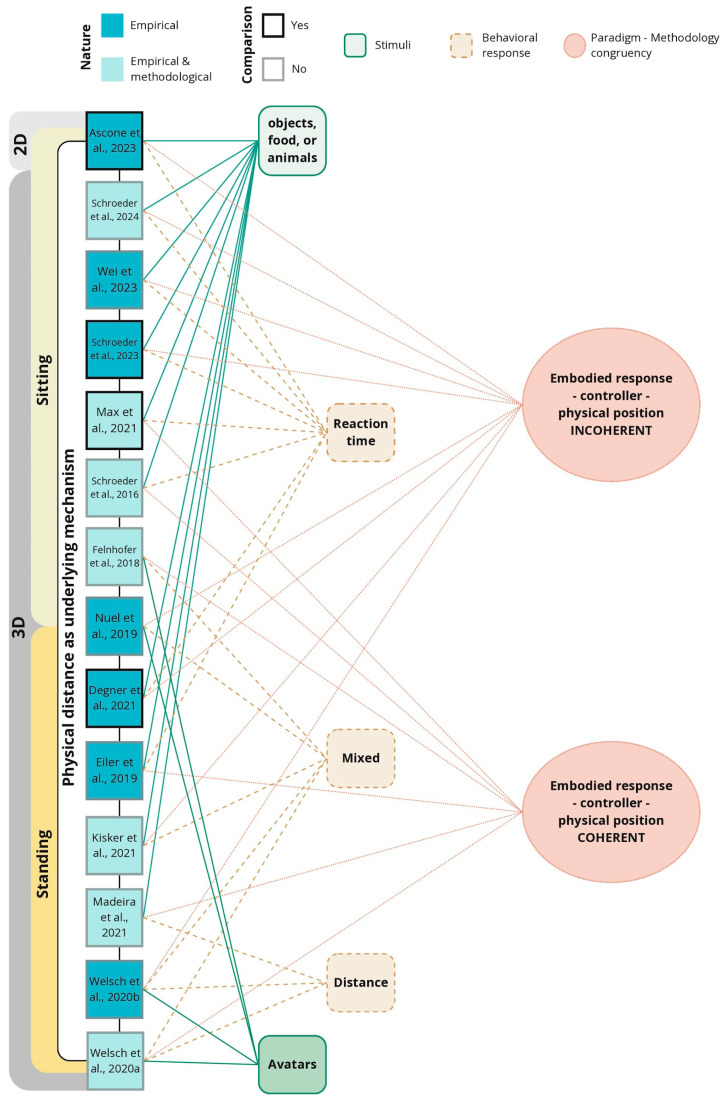
A conceptual map illustrating the relationship between all included articles (labeled by author and year), the types of stimuli (green), and the behavioral responses (orange) measured within the approach and avoidance bias research. From left to right, the map characterizes studies into empirical and methodological, distinguishing between 2D and 3D stimuli settings. It contrasts those studies that used small stimuli (such as objects, food, and animals) with those that promoted whole-body interaction with virtual avatars and associated types of behavioral responses measured. Paradigm–methodology congruence identifies whether each study’s embodied responses and devices used to mimic movements in virtual reality were coherent or incoherent with participants’ physical position. Lines denote the interactions of each study with the categories [1,4,45,46,47,48,49,50,51,52,53,54,55,56].

The benefits of understanding how an AAB occurs in natural dynamic environments are undeniable, particularly as interest in this area grows with state-of-the-art technologies. The ability to simulate realistic settings using VR opens up new avenues for exploring the AAB with greater ecological validity; however, this may also lead to less standardized methodological procedures. The interest in novel approaches to the AAB using VR and deviating from traditional paradigms has increased over the years (Figure 2). This shift has led to diverse methodologies and designs that illustrate complex interactions between experimental stimuli used and embodied responses measured. Figure 4 presents an organized overview of the studies in this review, clearly delineating those employing 2D versus 3D stimuli involving sitting or standing environments. Notably, most studies that address empirical and methodological dimensions utilized sophisticated stimuli, including virtual avatars and interactive objects, to elicit a broader range of behavioral responses. Although studies shared the fundamental assumption that changes in physical distance (increase and decrease) modulate the AAB [69], these changes were measured via distinct behavioral metrics. Traditional measures like reaction time were most common among studies addressing only empirical aspects of the AAB within sitting scenarios that facilitated the physical interactions with the virtual stimuli. Mixed and distance-related measurements collected as behavioral responses emphasized the influence of both real and virtual settings on participant behavior and were most often used within standing scenarios. Notably, most studies that used alternative approaches (e.g., standing scenarios, avatar interactions, and mixed or distance-related responses) aligned toward more coherent experimental designs, prioritizing the ecological niche of the behavioral element involved. The conceptual map illustrates a clear distinction between coherent and incoherent paradigms, indicating that, while some studies successfully aligned participants’ embodied responses in VR with their physical, real-world position (such as matching stepping actions in the VR environment with actual stepping while standing) and employed devices like motion-tracking cameras that preserved the integrity of the movement, other studies failed to maintain this coherency. This relational map highlights the necessity for future research to ensure coherent experimental setups to enhance the findings’ validity and applicability. In the future, it will be essential to prioritize methodological rigor when developing VR environments to achieve consistent and reliable measurements of the AAB, fostering more nuanced insights into the complex behavioral characteristics of the AAB.

A key finding derived from this relational examination of the different approaches to studying AAB using VR concerns the importance of coherent design paradigms and their methodological implementations. Eiler et al. [4] emphasize the need for stimuli with distinctive yet subtle features, allowing them to remain recognizable despite rapid automatic actions. Coherence between stimuli and participants’ physical movements, such as grasping and stepping, must be maintained to ensure meaningful interactions within the 3D space. For instance, if the AAB is assessed by measuring stepping behavior in VR, participants should be physically standing, with actual stepping movements captured, rather than using controller inputs. Similarly, grasping actions should be facilitated by actual hand movements rather than button presses. Solzbacher et al. [15] demonstrate that designs promoting such coherent interactions of, for example, pulling and pushing (using a joystick vs. a response pad) show a significant reaction time advantage, underscoring the critical bodily component of the AAB. Moreover, as Keshava et al. [70] show, realistic action affordances impact goal-oriented planning, meaning that experiments should preserve the integrity of physical and virtual movement affordances. Thus, experimental designs integrating coherent action simulation can provide deeper insights into the AAB’s underlying mechanisms and improve the effectiveness of therapeutic treatment.

However, this dichotomy between experimental design and afforded physical actions remains implicit to current AAB research. For instance, some bodily responses used to measure the AAB, such as engaging in verbal conversations [49] and tapping [48], deviate from the idea of an embodied component inherent to AAB that is rooted in its evolutionary role [6,15]. These movements seem disconnected from the actual AAB tendencies they aim to assess and are unlikely to activate the core behavioral mechanisms underlying the AAB. This misalignment suggests that tasks such as conversing and tapping may not effectively engage the intuitive and automatic action-oriented responses that the AAB is theorized to evoke. Instead, these tasks risk focusing on cognitive processing rather than eliciting genuine AAB tendencies. In cognitive-bias modification (CBM), Van Dessel et al.’s [71] ABC training (Antecedent–Behavioral Choice–Consequence training) simulates real-life situations to aid individuals in making goal-relevant behavioral choices (to reduce alcohol addiction) in response to antecedent cues and their action consequences. However, joystick-based pulling and pushing in ABC training does not accurately reflect real-life behaviors associated with the approach and avoidance of alcohol. The incoherence between the ABC training method and real-life behavior may lead to participants experiencing cognitive dissonance. If individuals are trained to push and pull images rather than engage in actions that mimic actual drinking behaviors, they may struggle to internalize the training, as they may not develop the necessary skills to handle actual situations involving alcohol consumption. Similarly, Markmann and Brendl’s [72] interpretation of the manikin task highlights how incoherent movements in experimental designs can obscure a proper understanding of the AAB. They show a compatibility effect that relies on participants’ spatial representation of themselves rather than on their physical actions. This misalignment between conceptual and physical actions may lead to results that do not genuinely reflect the AAB’s underlying mechanisms, emphasizing the need to align perceptual and motor tasks with participants’ spatial self-representations to maintain the integrity of action-relevant movements. Yet, using pushing and pulling movements to positive and negative words, which are not actions typically associated with such stimuli in real life, questions the reliability of the findings. Therefore, coherent experimental designs that preserve the integrity of physical and simulated movements would advance our understanding of the AAB in everyday life scenarios and, when possible, improve the reliability of physiological data collection.

### 4.4. Understanding the AAB Beyond the Distance Mechanism?

Our results align with the standard definition of the AAB as a distance-regulation mechanism. This implies that changes in the distance between an agent and a target object (i.e., increasing or decreasing) account for the behavioral phenomena underlying the bias [10,15,69]. Accordingly, pushing and pulling actions, often conceptualized as arm extension and flexion, are among the most frequently studied behaviors in tasks exploring the AAB in both desktop-based and VR setups [4,15,51,55,56]. However, framing pushing and pulling behaviors within the context of the AAB is not without criticism. One primary concern is the lack of a single, inherent meaning for these actions to be exclusively categorized as approach or avoidance behaviors [73,74,75]. For instance, retracting the hand toward the body (a pulling movement typically associated with approach) could instead signify avoidance, such as when withdrawing the hand from a heat source [50]. This is also possible when using walking towards (i.e., approach) or away (i.e., avoid) from an object as the behavioral action to study the bias. When only considering the reduction or increase in the distance, the agency of the subject is neglected to a secondary degree. For example, an agent might reduce the distance (considered as an approach behavior) between its own body and an insect (e.g., a spider) to capture or terminate it (an avoidance behavior). These examples illustrate how an action traditionally conceptualized as approach behavior (e.g., arm flexion, walking towards an object) can, in specific contexts, function as avoidance behavior [50]. This underscores the fact that the distance-regulation mechanism is highly context- and stimulus-dependent, a factor that must be considered when investigating the AAB.

On the other hand, these movements are limited in scope, as not all behaviors towards objects can simply be explained by the dichotomies of being pushed, pulled, or approached/avoided through walking towards or away. As Nuel and colleagues [50] argue, a comprehensive conceptualization of the AAB must account for the situated nature of the bias, recognizing the importance of the agent’s history of interactions and its available action possibilities. In this context, the agency of the organism and the affordances of the objects and environment (i.e., possibilities for interaction [76]) should be considered when studying the bias [77,78,79]. For example, this could involve expanding the range and variety of actions an agent can take when encountering a particular object. In this regard, VR offers researchers a unique opportunity to explore a range of simultaneous interaction options, moving beyond the traditional dichotomy, by observing the agent’s natural behavior as they interact with the presented stimuli.

### 4.5. Integrating (Neuro)Physiology into AAB Studies Using VR

The systematization of the literature shows a need for the inclusion of (neuro)physiological measurements when studying the AAB in VR. Of the fourteen reviewed articles, only two [46,53] incorporated physiological measurements, focusing on brain activity. This underscores a need for further exploration of (neuro)physiological dynamics in more ecologically valid settings related to the AAB. Notably, while studies on the (neuro)physiological dynamics of the AAB are relatively scarce, most studies exploring the (neuro)physiological dynamics are restricted to computer-based setups. Furthermore, stationary neuroimaging techniques are often used, with a clear tendency for systems that provide high-spatial-resolution data compared to others (e.g., functional magnetic resonance imaging; fMRI) [3,80,81]. This tendency aligns with the standard analysis previously conducted on the AAB, which is mainly related to activity in the amygdala due to its involvement in motivation and emotional responses [82,83,84]. The limited application of (neuro)physiological measurements in VR settings may stem from the challenges of integrating high spatial resolution neuroimaging technologies with immersive VR setups [85]. This presents an opportunity for further research to address these technological and methodological barriers.

The AAB has also been explored using other neuroimaging techniques. Frontal Alpha Asymmetry (FAA) is one of the most commonly implemented analyses when studying the AAB using electroencephalography (EEG) [86,87]. FAA has been primarily related to emotion, motivation, and cognitive control [88,89,90], which are the most common underlying cognitive processes associated with the AAB [75,91,92]. This line of research shows the feasibility of studying the AAB using techniques with lower spatial resolution and higher temporal resolution. Furthermore, it opens up the possibility of studying the AAB using technical–methodological frameworks, such as mobile brain/body imaging (MoBI; [93,94,95]), which allows the study of brain dynamics during mobile tasks involving more naturalistic approach–avoidance scenarios. Hence, studying the AAB in more naturalistic conditions using VR or in real-life setups posits a complementary approach to classical laboratory setups, allowing for the development of tasks in real-world contexts (e.g., simulated or real situations) where individuals have higher freedom for self-agency and interaction [96,97,98,99,100,101,102]. This approach will allow for the exploration of common EEG markers associated with the AAB, such as FAA, in a more naturalistic and meaningful way as the bias occurs in real life.

### 4.6. Critical Challenges and Opportunities for Advancement on Studying the AAB in VR

The reported challenges and limitations of the included studies are relatively underexplored. Most of the included articles report participant-specific limitations related to using VR for experiments (i.e., ethical aspects, habituation, motion sickness, among others) and limitations regarding stimulus type and its characteristics. However, these discussions often overlook the pivotal technical and methodological considerations for transferring the AAB into more naturalistic setups. For example, Eiler et al. [27] provide a thorough analysis of critical aspects to consider for such translation, including high costs, the need for technological expertise, equipment constraints, and challenges in terms of data accuracy. A comprehensive examination of not only conceptual but also technical and methodological challenges is needed to foster reproducibility across multiple populations and research settings. Furthermore, this can encourage the development of innovative approaches that advance the field. Additionally, given the diversity of topics addressed when studying the AAB in VR, establishing a standardized methodological framework could create consistency across studies and enable researchers to build upon one another’s work.

Finally, considering future directions for studying the AAB in VR, many included studies emphasize the need to address embodied aspects of virtual environments to better replicate reality. Also, integrating (neuro)physiological measurements could provide valuable insights into the neural and motor processes underlying the AAB. This approach would allow researchers to explore its (neuro)physiological dynamics in naturalistic interactions, bridging the gap between experimental findings and real-world applications. Furthermore, exploring the AAB in real-life scenarios by placing participants in novel and coherent setups could enhance ecological validity and offer a more nuanced understanding of how VR findings translate to everyday behaviors. Balancing such advancements with scientific rigor and methodological novelty will ensure that research remains robust and reproducible while pushing the boundaries of what can be achieved in this field.

### 4.7. General Recommendations for Translating the AAB into VR

The results from the present review show a strong tendency to translate the AAB into more naturalistic setups, whether to study the bias itself or for applications such as training or therapeutic interventions. However, when it comes to understanding the bias, the field remains diverse in its research questions and the methodological approaches employed. The following sections will present general recommendations for consistently translating the AAB into VR.

Several of the desktop-based AATs use stimuli that come from databases that have been standardized. Examples of this are the use of pictures from the International Affective Picture System (IAPS; [103]), the Nencki Affective Picture System (NAPS; [104]), or the Image Stimuli for Emotion Elicitation (ISEE; [105]), among others. Using stimuli from these datasets ensures that they have been rigorously tested and validated for eliciting specific emotional states (e.g., positive or negative affect). However, as experimental setups transition to more naturalistic environments, 3D stimuli are often preferred because they provide a more immersive and realistic experience, closely mirroring real-world conditions. Nevertheless, the adequacy of the stimuli to generate the phenomenon that we aim to find, such as the AAB, must remain a critical consideration [106]. To address this, we propose two complementary strategies. First, a desktop-based control condition will be implemented to prove the adequacy of the stimuli in generating the AAB. Some of the reviewed studies that included a control condition (e.g., desktop-based) used screenshots from the VR stimuli in a classical AAT to assess the adequacy of the stimuli without any potential cofounders generated by the VR setup. Moreover, this approach facilitates a systematic examination of the bias, transitioning from controlled experimental setups to more complex, naturalistic environments that closely simulate real-world scenarios [107]. This progression enhances the findings’ ecological validity, relevance, and applicability, bridging the gap between laboratory research and practical, real-world applications. Secondly, it is recommended that validated assessment tools be employed to evaluate key dimensions of the stimuli before their implementation in VR. An example is using the Self-Assessment Mannequin (SAM; [108,109,110]), which can measure dimensions such as pleasure, arousal, and dominance. These measures can help rule out potential confounding factors and ensure the stimuli’s characteristics align with experimental goals. Combining these approaches can enhance confidence in the stimuli’s efficacy while maintaining the rigor of the research, even when the specific stimuli do not belong to a standardized database.

Another essential consideration when designing VR experiments is the type of gesture implemented. Even when a virtual environment already provides a more naturalistic setup, in terms of the stimuli presented, achieving a closer approximation to real-life phenomena requires careful attention to the range of actions participants can perform. Research highlights that the type of embodied response generated significantly influences the phenomena under study [15,70]. Therefore, incorporating coherent interaction between the actions the subject performs inside and outside the VR setup is crucial. For example, suppose that inside the VR environment, the participant has the experience of getting closer to or further away from a target stimulus or avatar by walking. In that case, we encourage the design of experiments where the participants are able to perform these movements outside of the environment (e.g., physically stepping forward and backward) instead of using a joystick to control the movement. By incorporating coherent embodied actions that enhance ecological validity and do not artificially restrict the agents’ movements in their interaction with the stimuli, we can assess how affordances for action impact the AAB, providing deeper insights into the underlying mechanisms.

In order to achieve this goal, technical and methodological aspects must be assessed. First, it is important to consider what type of devices are used to record behavioral movements. Following the findings of our review, we recommend prioritizing motion sensors over traditional input devices, like joysticks or response pads, to enhance naturalistic actions since they present fewer restrictions for actions [111,112]. Secondly, graphical features of the virtual environment are equally critical as they contribute to the immersive and embodied experience. For instance, in scenarios involving stepping forward and backward, the environment should dynamically adjust accordingly to the performed movement in order to simulate as much as possible the real-world experience (e.g., realistic object scaling, perspective shifts, detail levels). Similarly, for grasping behaviors, the design should account for interactive elements, such as manipulating virtual objects, ensuring that the experience mirrors real-world tactile interactions. By addressing these technical and methodological aspects, we can enhance the implementation of naturalistic actions, leading to a better understanding of the embodied mechanisms of the AAB in the real world.

Finally, we strongly advocate for the collection of multimodal data to enhance the understanding of the underlying mechanisms of biases. Although only two reviewed articles include some type of (neuro)physiological data, its incorporation might provide insights into the foundational processes driving the AAB. For example, it would be possible to explore and understand the role of autonomic physiological states in the decision-making process [19], stress [113], and other dynamics. As we stated before, we recommend formulating research questions targeting this type of data since it will significantly contribute to understanding the basis mechanism of the bias. In this sense, we encourage the development of innovative research questions involving (neuro)physiological dynamics underlying the AAB. Such an approach will facilitate the inclusion of multimodal data, such as eye tracking, EEG, and even other measures such as galvanic skin response (GSR) and ECG, for a comprehensive understanding of AAB.

## 5. Conclusions

This systematic review underscores the potential of VR as a promising ecological framework for studying the AAB, enabling dynamic and immersive interactions. Our findings reveal diverse research questions and methodologies that leverage VR to explore and better understand the AAB. However, we highlight the pressing need for a well-structured framework to systematically investigate AAB tendencies within VR environments. Addressing foundational challenges—such as developing baseline principles for VR-based designs in naturalistic contexts—is essential for advancing both the scientific understanding of AAB and its practical applications. By tackling these challenges, researchers can establish more robust and reproducible experimental paradigms and design effective interventions to help individuals recognize and modify their biases, ultimately fostering more equitable and balanced behaviors.

## Figures and Tables

**Table 1:** This table outlines the PICO (population, intervention, comparison, outcome) criteria applied during the screening process to guide the selection of articles for inclusion.

PICO Criteria for the Inclusion of Articles in the Review	
Criteria	Determinants
Population	Human participants, including clinical populations and healthy subjects
Intervention	All types of experimental paradigms are oriented to studying the approach–avoidance bias using virtual reality or a natural setup. This review does not include using VR or a natural setup as training or treatment.
Comparison	The presence of a comparison or control condition was not required to be included in the review.
Outcome	Any type of outcome was expected from the included articles regarding the study of the approach–avoidance bias in virtual reality.
Others	No timing restrictions; virtual reality and natural setups; studies reported in English; published studies in peer-reviewed journals.

**Table 2:** Detailed risk of bias assessment results for each included article.

Risk of Bias Assessment										
*Case–Control Studies* ^1^										
Reference	Q1	Q2	Q3	Q4	Q5	Q6	Q7	Q8	Q9	Q10
Felnhofer et al., 2018 [49]	Yes	Yes	Yes	Yes	Yes	Yes	Yes	Yes	Not Applicable	Yes
Eiler et al., 2019 [4]	Yes	Yes	Yes	Yes	Yes	Unclear	Unclear	Yes	Not Applicable	Yes
Degner et al., 2021 [1]	Yes	Yes	Yes	Yes	Yes	Unclear	Unclear	Yes	Not Applicable	Yes
Max et al., 2021 [46]	Yes	Yes	Yes	Yes	Yes	Unclear	Unclear	Yes	Not Applicable	Yes
Schroeder et al., 2023 [47]	Yes	Yes	Yes	Yes	Yes	Yes	Yes	Yes	Not Applicable	Yes
Ascone et al., 2023 [56]	Yes	Yes	Yes	Yes	Yes	Unclear	Unclear	Yes	Not Applicable	Yes
Schroeder et al., 2024 [48]	Yes	Yes	Yes	Yes	Yes	Yes	Yes	Yes	Not Applicable	Yes
*Cross-Sectional Studies* ^2^										
Reference	Q1	Q2	Q3	Q4	Q5	Q6	Q7	Q8		
Schroeder et al., 2016 [50]	Yes	Yes	Yes	Yes	Unclear	Unclear	Yes	Yes		
Nuel et al., 2019 [51]	Yes	Yes	Yes	Yes	Yes	Yes	Yes	Yes		
Welsch et al., 2020 [51]	Yes	Yes	Yes	Yes	Yes	Yes	Yes	Yes		
Welsch et al., 2020 [52]	Yes	Yes	Yes	Yes	Yes	Yes	Yes	Yes		
Kisker et al., 2021 [53]	Yes	Yes	Yes	Yes	Yes	Yes	Yes	Yes		
Madeira et al., 2021 [54]	Yes	Yes	Yes	Yes	Unclear	Unclear	Yes	Yes		
Wei et al., 2023 [55]	Yes	Yes	Yes	Yes	Yes	Yes	Yes	Yes		

^1^ Q1: Were the groups comparable other than the presence of disease in cases or the absence of disease in controls? Q2: Were cases and controls matched appropriately? Q3: Were the same criteria used for the identification of cases and controls? Q4: Was exposure measured in a standard, valid, and reliable way? Q5: Was exposure measured in the same way for cases and controls? Q6: Were confounding factors identified? Q7: Were strategies to deal with confounding factors stated? Q8: Were outcomes assessed in a standard, valid, and reliable way for cases and controls? Q9: Was the exposure period of interest long enough to be meaningful? Q10: Was appropriate statistical analysis used? ^2^ Q1: Were the criteria for inclusion in the sample clearly defined? Q2: Were the study subjects and the setting described in detail? Q3: Was the exposure measured validly and reliably? Q4: Were objective, standard criteria used for measurement of the condition? Q5: Were confounding factors identified? Q6: Were strategies to deal with confounding factors stated? Q7: Were the outcomes measured validly and reliably? Q8: Was appropriate statistical analysis used?

**Table 3:** Detailed description of the experimental designs implemented in the reviewed studies and the variables of interest.

Reference	Experimental Paradigm
Schroeder et al., 2016 [45]	Approach–avoidance task to measure the interaction with food and ball objects
Felnhofer et al., 2018 [49]	Interactional task with agents
Nuel et al., 2019 [50]	E1 and E2: Approach–avoidance task to agents in different contexts
Eiler et al., 2019 [4]	Sorting task to measure interaction between smoke and non-smoke related cues
Welsch et al., 2020 [51]	E1: Distance reduction task to agents of different genders and facial expressions; E2 and E3: Approach–Avoidance Task regarding agents of different genders
Welsch et al., 2020 [52]	E1: Approach–avoidance task to agents of different gender; E2: Distance reduction task to agents of different genders.
Degner et al., 2021 [1]	Approach–avoidance task to measure the interaction between butterflies and spiders
Kisker et al., 2021 [53]	Cave exploration
Madeira et al., 2021 [54]	Elevated Plus-Maze
Max et al., 2021 [46]	Decision-making task regarding food and non-food items
Wei et al., 2023 [55]	Approach–avoidance task to measure the interaction between game-related and neutral cues
Schroeder et al., 2023 [47]	Stop-signal task to measure food-specific inhibitory control
Ascone et al., 2023 [56]	Approach–avoidance task to measure the interaction between alcoholic and non-alcoholic beverages.
Schroeder et al., 2024 [48]	Stop-signal task to measure foo-specific inhibitory control

E = Experiment.

**Table 4:** Detailed description of paradigm-related characteristics reported in the included studies.

Reference	Trial Number	Stimuli	Embodied Response	Behavioral Data	Comparison
Schroeder et al., 2016 [45]	240	3D; food and nonfood ball objects	Grabbing and warding off	Reaction time	No
Felnhofer et al., 2018 [49]	N/A	3D; virtual agents	Verbal interaction	Type of reaction	No
Nuel et al., 2019 [50]	E1: 30 E2: 30	3D; virtual agents	Leaning and stepping forward–backward	Type of reaction	No
Eiler et al., 2019 [4]	20	3D; smoke-related and non-related	Pushing and pulling	Reaction time	No
Welsch et al., 2020 [51]	E1: 40 E2: 160 E3: 160	3D; virtual agents	E1: Walking; E2 and E3: Pushing and pulling	Reaction time, type of reaction, and velocity	No
Welsch et al., 2020 [52]	E1: 160 E2: 40	3D; virtual agents	E1: Step forward–backward; E2: Walking	Reaction time, type of reaction, and velocity	No
Degner et al., 2021 [1]	128	3D; plants, butterflies, and spiders	Stepping forward–backward	Reaction time and position	Yes
Kisker et al., 2021 [53]	N/A	3D; Cave	Exploring (walking)	Exploration time and type of reaction	No
Madeira et al., 2021 [54]	N/A	3D; E1: Elevated Plus Maze; E2: Modified EPM	Exploring (walking)	Exploration time, type of reaction, and distance walked	No
Max et al., 2021 [46]	192	3D; balls, food, and office objects	Grabbing and locating objects	Reaction time	Yes
Wei et al., 2023 [55]	96	3D; game-related and non-game cartoons	Pushing and pulling	Reaction time	No
Schroeder et al., 2023 [47]	400	3D; chocolate and similar objects	Grabbing	Reaction time and position	Yes
Ascone et al., 2023 [56]	156	2D; alcoholic and non-alcoholic drinks	Pushing and Pulling; Grabbing	Reaction time	Yes
Schroeder et al., 2024 [48]	200	3D; food and shoes	Tapping	Reaction time	No

N/A: not available; E = experiment.

**Table 5:** Detailed description of technical characteristics reported in the included studies.

Reference	VR System	Resolution	Sampling Rate	Controllers	Development System	Physiology	Position—Movement
Schroeder et al., 2016 [45]	Oculus Rift, HMD	N/A	N/A	Motion sensors	VR API, Unity 3D	No	Sitting, arm and hand movements
Felnhofer et al., 2018 [49]	Sony HMZ-T1, HMD	N/A	N/A	TrackIR 5, head tracking system; HTC Desire SV smartphone	GIMP, OGRE 3D	No	Sitting
Nuel et al., 2019 [50]	HTC Vive, HMD	N/A	N/A	HTC controllers	N/A	No	E1: Sitting; leaning back; E2: Standing; stepping
Eiler et al., 2019 [4]	HTC Vive HMD	2160 × 1200 pixels	90 Hz	Motion sensor and HTC Vive controllers	Unity 3D	No	Standing
Welsch et al., 2020 [51]	LCD shutter glasses	1400 × 1050 pixels	60 Hz	Motion sensor	Vizard 5	No	E1: Standing, walking; E2 and E3: Standing, moving a joystick with the arm
Welsch et al., 2020 [52]	HTC Vive HMD	1080 × 1200 pixels	90 Hz	HTC Vive controller	Vizard 5	No	Standing, stepping
Degner et al., 2021 [1]	HTC Vive Pro, HMD	1440 × 1600 pixels	90 Hz	HTC Vive Controller	Unity	No	Standing, stepping
Kisker et al., 2021 [53]	HTC Vive Pro, HMD	N/A	N/A	HTC Vive controller	Unity	EEG and ECG	Standing, walking
Madeira et al., 2021 [54]	Infitec Premium color-filtering glasses	1920 × 1200 pixels	60 Hz	Motion sensor, Xbox 360 controller	CS-Research VTplus	No	Standing, walking
Max et al., 2021 [46]	Oculus Rift CV1, HMD	1080 × 1200 pixels	N/A	Motion sensor	Unity	fNIRS	Sitting, arm and hand movements
Wei et al., 2023 [55]	HTC Vive HMD	N/A	N/A	N/A	N/A	No	Sitting, arm and hand movements
Schroeder et al., 2023 [47]	HTC Vive, HMD	N/A	120 Hz	HTC Vive Wand controller	Unity	No	Sitting, hand movements
Ascone et al., 2023 [56]	HTC Vive Pro, HMD	2880 × 1660 pixels	N/A	VR-controller	N/A	No	Sitting, arm and hand movements
Schroeder et al., 2024 [48]	Meta Quest 2, HMD	N/A	62 Hz	Meta Quest 2 controller	N/A	No	Sitting, hand movements

N/A: not available; E = experiment.

**Table 6:** Summary of limitations and proposed future directions for using VR to study the AAB, as identified in the included articles.

Reference	Reported Limitations	Suggested Future Directions
Schroeder et al., 2016 [45]	Lower-level features of the stimuli might be involved in the bias.	To examine movement and speed trajectories; To investigate lower-level features.
Felnhofer et al., 2018 [49]	Participants’ previous experience in VR was motion sickness.	To consider virtual social environments’ ecological validity and explore specific disorder-dependent social behaviors towards avatars in different populations.
Nuel et al., 2019 [50]	The feeling of presence: engagement with the VR environment in comparison with the real-world	To explore the ecological operationalizations of approach–avoidance behaviors, suggesting that more extensive behavioral repetition and specific contingencies may be necessary
Eiler et al., 2019 [4]	N/A	To improve experimental conditions for feeling of presence; To use more realistic graphics in VR; To improve reaction time measurements; To implement full-body tracking to increase immersion and embodiment
Welsch et al., 2020 [51]	N/A	To improve the ecological validity of the VR setup
Welsch et al., 2020 [52]	Length of the experiment and habituation	To test the reliability and validity of the AAB in VR.
Degner et al., 2021 [1]	Habituation, strategy development, and physical demand; Stimuli type	To estimate the optimal trial number that allows reliable measurement without compromising observed effects and counterbalance conditions; To implement full-body tracking as a target measure for analysis
Kisker et al., 2021 [53]	Impact of movement on physiological measures; Ethical challenges of using VR	To research whether factors other than emotion and immersion may have varying effects on the dimensions of presence
Madeira et al., 2021 [54]	Lack of psychophysiological measures	To assess other behavioral measures; To generate naturalistic EPM environments to increase validity
Max et al., 2021 [46]	N/A	To improve the ecological validity of the VR setup
Wei et al., 2023 [55]	Stimuli type	N/A
Schroeder et al., 2023 [47]	Stimuli type	N/A
Ascone et al., 2023 [56]	N/A	To enhance the grasping component, To test different VR setups, To use more realistic stimuli
Schroeder et al., 2024 [48]	N/A	To manipulate stimuli modality

N/A = not available.

## Data Availability

Data are available through Open Science Framework: https://osf.io/huv6x/.

## Supplementary Materials

The following supporting information can be downloaded at https://www.mdpi.com/article/10.3390/brainsci15020103/s1: File S1. String of search for Web of Science; File S2. Data items for data extraction.

## Abbreviations

The following abbreviations are used in this manuscript:

2D	Two-dimensional
3D	Three-dimensional
AAB	Approach and Avoidance Bias
AAT	Approach–Avoidance Task
AIB	Automatic Implicit Bias
CBM	Cognitive-Bias Modification
ECG	Electrocardiogram
EEG	Electroencephalogram
FAA	Frontal Alpha Asymmetry
fNIRS	Functional Near Infrared Spectroscopy
GSR	Galvanic Skin Response
HMD	Head-Mounted Display
IAPS	International Affective Picture System
ISEE	Image Stimuli for Emotion Elicitation
JBI	Joanna Briggs Institute
MoBI	Mobile Brain/Body Imaging
NAPS	Nencki Affective Picture System
PRISMA	Preferred Reporting Items for Systematic Reviews and Meta-analyses
SAM	Self-Assessment Mannequin
SRC	Stimulus–Response Compatibility
VR	Virtual Reality

## References

[1] Degner J., Steep L., Schmidt S., Steinicke F. (2021). Assessing Automatic Approach-Avoidance Behavior in an Immersive Virtual Environment. Front. Virtual Real..

[2] McNaughton N., DeYoung C.G., Corr P.J., Absher J.R., Cloutier J. (2016). Chapter 2—Approach/Avoidance. Neuroimaging Personality, Social Cognition, and Character.

[3] Aupperle R.L., Melrose A.J., Francisco A., Paulus M.P., Stein M.B. (2015). Neural Substrates of Approach-Avoidance Conflict Decision-Making. Hum. Brain Mapp..

[4] Eiler T.J., Grünewald A., Machulska A., Klucken T., Jahn K., Niehaves B., Gethmann C.F., Brück R. (2019). A Preliminary Evaluation of Transferring the Approach Avoidance Task into Virtual Reality. Proceedings of the Information Technology in Biomedicine.

[5] Kenrick D.T., Shiota M.N., Elliot A.J. (2008). Approach and Avoidance Motivation(s): An Evolutionary Perspective. Handbook of Approach and Avoidance Motivation.

[6] Fridland E., Wiers C.E. (2018). Addiction and Embodiment. Phenomenol. Cogn. Sci..

[7] Lang P.J. (1995). The Emotion Probe. Studies of Motivation and Attention. Am. Psychol..

[8] Loijen A., Vrijsen J.N., Egger J.I.M., Becker E.S., Rinck M. (2020). Biased Approach-Avoidance Tendencies in Psychopathology: A Systematic Review of Their Assessment and Modification. Clin. Psychol. Rev..

[9] Elliot A.J. (2006). The Hierarchical Model of Approach-Avoidance Motivation. Motiv. Emot..

[10] Krieglmeyer R., De Houwer J., Deutsch R. (2013). On the Nature of Automatically Triggered Approach–avoidance Behavior. Emot. Rev..

[11] Phaf R.H., Mohr S.E., Rotteveel M., Wicherts J.M. (2014). Approach, Avoidance, and Affect: A Meta-Analysis of Approach-Avoidance Tendencies in Manual Reaction Time Tasks. Front. Psychol..

[12] Zech H.G., Rotteveel M., van Dijk W.W., van Dillen L.F. (2020). A Mobile Approach-Avoidance Task. Behav. Res. Methods.

[13] Chen M., Bargh J.A. (1999). Consequences of Automatic Evaluation: Immediate Behavioral Predispositions to Approach or Avoid the Stimulus. Personal. Soc. Psychol. Bull..

[14] Czeszumski A., Albers F., Walter S., König P. (2021). Let Me Make You Happy, and I’ll Tell You How You Look Around: Using an Approach-Avoidance Task as an Embodied Emotion Prime in a Free-Viewing Task. Front. Psychol..

[15] Solzbacher J., Czeszumski A., Walter S., König P. (2022). Evidence for the Embodiment of the Automatic Approach Bias. Front. Psychol..

[16] Kaspar K., Gameiro R.R., König P. (2015). Feeling Good, Searching the Bad: Positive Priming Increases Attention and Memory for Negative Stimuli on Webpages. Comput. Human Behav..

[17] Kaspar K., Hloucal T.-M., Kriz J., Canzler S., Gameiro R.R., Krapp V., König P. (2013). Emotions’ Impact on Viewing Behavior under Natural Conditions. PLoS ONE.

[18] Ferrari G.R.A., Möbius M., Becker E.S., Spijker J., Rinck M. (2018). Working Mechanisms of a General Positivity Approach-Avoidance Training: Effects on Action Tendencies as Well as on Subjective and Physiological Stress Responses. J. Behav. Ther. Exp. Psychiatry.

[19] Livermore J.J.A., Klaassen F.H., Bramson B., Hulsman A.M., Meijer S.W., Held L., Klumpers F., de Voogd L.D., Roelofs K. (2021). Approach-Avoidance Decisions Under Threat: The Role of Autonomic Psychophysiological States. Front. Neurosci..

[20] Grossman P., Taylor E.W. (2007). Toward Understanding Respiratory Sinus Arrhythmia: Relations to Cardiac Vagal Tone, Evolution and Biobehavioral Functions. Biol. Psychol..

[21] Laborde S., Mosley E., Thayer J.F. (2017). Heart Rate Variability and Cardiac Vagal Tone in Psychophysiological Research—Recommendations for Experiment Planning, Data Analysis, and Data Reporting. Front. Psychol..

[22] Porges S.W., Doussard-Roosevelt J.A., Maiti A.K. (1994). Vagal Tone and the Physiological Regulation of Emotion. Monogr. Soc. Res. Child Dev..

[23] Holleman G.A., Hooge I.T.C., Kemner C., Hessels R.S. (2021). The Reality of “Real-Life” Neuroscience: A Commentary on Shamay-Tsoory and Mendelsohn (2019). Perspect. Psychol. Sci..

[24] Kothe C., Shirazi S.Y., Stenner T., Medine D., Boulay C., Grivich M.I., Mullen T., Delorme A., Makeig S. (2024). The Lab Streaming Layer for Synchronized Multimodal Recording. bioRxiv.

[25] Petukhov I.V., Glazyrin A.E., Gorokhov A.V., Steshina L.A., Tanryverdiev I.O. (2020). Being Present in a Real or Virtual World: A EEG Study. Int. J. Med. Inform..

[26] Fadeev K.A., Smirnov A.S., Zhigalova O.P., Bazhina P.S., Tumialis A.V., Golokhvast K.S. (2020). Too Real to Be Virtual: Autonomic and EEG Responses to Extreme Stress Scenarios in Virtual Reality. Behav. Neurol..

[27] Eiler T.J., Grünewald A., Wahl M., Brück R. (2020). AAT Meets Virtual Reality. Proceedings of the Computer Vision, Imaging and Computer Graphics Theory and Applications.

[28] Eiler T.J., Grünewald A., Brück R. Fighting Substance Dependency Combining AAT Therapy and Virtual Reality with Game Design Elements. Proceedings of the 14th International Joint Conference on Computer Vision, Imaging and Computer Graphics Theory and Applications (VISIGRAPP 2019).

[29] Jones E.B., Sharpe L. (2017). Cognitive Bias Modification: A Review of Meta-Analyses. J. Affect. Disord..

[30] Machulska A., Eiler T.J., Kleinke K., Grünewald A., Brück R., Jahn K., Niehaves B., Klucken T. (2021). Approach Bias Retraining through Virtual Reality in Smokers Willing to Quit Smoking: A Randomized-Controlled Study. Behav. Res. Ther..

[31] Santos W.M.D., Secoli S.R., Püschel V.A. (2018). de A. The Joanna Briggs Institute Approach for Systematic Reviews. Rev. Lat. Am. Enfermagem.

[32] Page M.J., McKenzie J.E., Bossuyt P.M., Boutron I., Hoffmann T.C., Mulrow C.D., Shamseer L., Tetzlaff J.M., Akl E.A., Brennan S.E. (2021). The PRISMA 2020 Statement: An Updated Guideline for Reporting Systematic Reviews. Rev. Esp. Cardiol..

[33] Moher D., Liberati A., Tetzlaff J., Altman D.G., Group P. (2010). Others Preferred Reporting Items for Systematic Reviews and Meta-Analyses: The PRISMA Statement. Int. J. Surg..

[34] Chandra K., Slater B., Ma M. (2023). Research Rabbit. https://www.researchrabbit.ai/.

[35] Tarnavsky-Eitan A., Smolyansky E., Knaan-Harpaz I., Perets S. (2019). Connected Papers. https://connectedpapers.com.

[36] van de Schoot R., de Bruin J., Schram R., Zahedi P., de Boer J., Weijdema F., Kramer B., Huijts M., Hoogerwerf M., Ferdinands G. (2021). An Open Source Machine Learning Framework for Efficient and Transparent Systematic Reviews. Nat. Mach. Intell..

[37] Munn Z., Barker T.H., Moola S., Tufanaru C., Stern C., McArthur A., Stephenson M., Aromataris E. (2020). Methodological Quality of Case Series Studies: An Introduction to the JBI Critical Appraisal Tool. JBI Evid. Synth..

[38] Barker T.H., Hasanoff S., Aromataris E., Stone J., Leonardi-Bee J., Sears K., Habibi N., Klugar M., Tufanaru C., Moola S. (2024). The Revised JBI Critical Appraisal Tool for the Assessment of Risk of Bias for Cohort Studies. JBI Evid. Synth..

[39] Popay J., Roberts H., Sowden A., Petticrew M., Arai L., Rodgers M., Britten N., Roen K., Duffy S. (2006). Guidance on the Conduct of Narrative Synthesis in Systematic Reviews: A product from the ESRC Methods Programme Version.

[40] Soni K.D. (2023). When Do We Need to Do Meta-Analysis!!!. Indian J. Anaesth..

[41] Field A.P., Gillett R. (2010). How to Do a Meta-Analysis. Br. J. Math. Stat. Psychol..

[42] Ratliff K.A., Smith C.T. (2022). Implicit Bias as Automatic Behavior. Psychol. Inq..

[43] van Nunspeet F., Ellemers N., Derks B. (2015). Reducing Implicit Bias: How Moral Motivation Helps People Refrain from Making “automatic” Prejudiced Associations. Transl. Issues Psychol. Sci..

[44] Payne B.K., Hannay J.W. (2021). Implicit Bias Reflects Systemic Racism. Trends Cogn. Sci..

[45] Schroeder P.A., Lohmann J., Butz M.V., Plewnia C. (2016). Behavioral Bias for Food Reflected in Hand Movements: A Preliminary Study with Healthy Subjects. Cyberpsychol. Behav. Soc. Netw..

[46] Max S.M., Schroeder P.A., Blechert J., Giel K.E., Ehlis A.-C., Plewnia C. (2021). Mind the Food: Behavioural Characteristics and Imaging Signatures of the Specific Handling of Food Objects. Brain Struct. Funct..

[47] Schroeder P.A., Mayer K., Wirth R., Svaldi J. (2023). Playing with Temptation: Stopping Abilities to Chocolate Are Superior, but Also More Extensive. Appetite.

[48] Schroeder P.A., Collantoni E., Meregalli V., Rabarbari E., Simonazzi C., Svaldi J., Cardi V. (2024). Persistent Avoidance of Virtual Food in Anorexia Nervosa-Restrictive Type: Results from Motion Tracking in a Virtual Stopping Task. Int. J. Eat. Disord..

[49] Felnhofer A., Kafka J.X., Hlavacs H., Beutl L., Kryspin-Exner I., Kothgassner O.D. (2018). Meeting Others Virtually in a Day-to-Day Setting: Investigating Social Avoidance and Prosocial Behavior towards Avatars and Agents. Comput. Hum. Behav..

[50] Nuel I., Fayant M.-P., Alexopoulos T. (2019). “Science Manipulates the Things and Lives in Them”: Reconsidering Approach-Avoidance Operationalization through a Grounded Cognition Perspective. Front. Psychol..

[51] Welsch R., von Castell C., Hecht H. (2020). Interpersonal Distance Regulation and Approach-Avoidance Reactions Are Altered in Psychopathy. Clin. Psychol. Sci..

[52] Welsch R., von Castell C., Rettenberger M., Turner D., Hecht H., Fromberger P. (2020). Sexual Attraction Modulates Interpersonal Distance and Approach-Avoidance Movements towards Virtual Agents in Males. PLoS ONE.

[53] Kisker J., Lange L., Flinkenflügel K., Kaup M., Labersweiler N., Tetenborg F., Ott P., Gundler C., Gruber T., Osinsky R. (2021). Authentic Fear Responses in Virtual Reality: A Mobile EEG Study on Affective, Behavioral and Electrophysiological Correlates of Fear. Front. Virtual Real..

[54] Madeira O., Gromer D., Latoschik M.E., Pauli P. (2021). Effects of Acrophobic Fear and Trait Anxiety on Human Behavior in a Virtual Elevated Plus-Maze. Front. Virtual Real..

[55] Wei W., Wang Q., Ding R., Dong R., Ni S. (2023). Playing Closer: Using Virtual Reality to Measure Approach Bias of Internet Gaming Disorder. Behav. Sci..

[56] Ascone L., Wirtz J., Mellentin A.I., Kugler D., Bremer T., Schadow F., Hoppe S., Jebens C., Kühn S. (2023). Transferring the Approach Avoidance Task into Virtual Reality: A Study in Patients with Alcohol Use Disorder versus Healthy Controls. Virtual Real..

[57] Jahn K., Oschinsky F.M., Kordyaka B., Machulska A., Eiler T.J., Gruenewald A., Klucken T., Brueck R., Gethmann C.F., Niehaves B. (2022). Design Elements in Immersive Virtual Reality: The Impact of Object Presence on Health-Related Outcomes. Internet Res..

[58] Smeijers D., Bulten E.H., Verkes R.-J., Koole S.L. (2021). Testing the Effects of a Virtual Reality Game for Aggressive Impulse Management: A Preliminary Randomized Controlled Trial among Forensic Psychiatric Outpatients. Brain Sci..

[59] Banakou D., Hanumanthu P.D., Slater M. (2016). Virtual Embodiment of White People in a Black Virtual Body Leads to a Sustained Reduction in Their Implicit Racial Bias. Front. Hum. Neurosci..

[60] Kishore S., Spanlang B., Iruretagoyena G., Szostak D., Slater M. (2021). A Virtual Reality Embodiment Technique to Enhance Helping Behavior of Police towards a Victim of Police Racial Aggression. PRESENCE Virtual Augment. Real..

[61] Peck T.C., Good J.J., Seitz K. (2021). Evidence of Racial Bias Using Immersive Virtual Reality: Analysis of Head and Hand Motions during Shooting Decisions. IEEE Trans. Vis. Comput. Graph..

[62] You C., Peck T., Stuart J., Gomes de Siqueira A., Lok B. (2024). What My Bias Meant for My Embodiment: An Investigation on Virtual Embodiment in Desktop-Based Virtual Reality. Front. Virtual Real..

[63] Persky S., Hollister B.M., Martingano A.J., Dolwick A.P., Telaak S.H., Schopp E.M., Bonham V.L. (2024). Assessing Bias toward a Black or White Simulated Patient with Obesity in a Virtual Reality-Based Genomics Encounter. CyberpsychologyBehav. Soc. Netw..

[64] Ferdous S.M.S., Michael A., Chowdhury T.I., Quarles J. Use of Scaling to Improve Reach in Virtual Reality for People with Parkinson’s Disease. Proceedings of the 2022 IEEE 10th International Conference on Serious Games and Applications for Health(SeGAH).

[65] Kampmann I.L., Emmelkamp P.M.G., Morina N. (2018). Does Exposure Therapy Lead to Changes in Attention Bias and Approach-Avoidance Bias in Patients with Social Anxiety Disorder?. Cognit. Ther. Res..

[66] Scherer K.R., Moors A. (2019). The Emotion Process: Event Appraisal and Component Differentiation. Annu. Rev. Psychol..

[67] Moors A., Fischer M. (2019). Demystifying the Role of Emotion in Behaviour: Toward a Goal-Directed Account. Cogn. Emot..

[68] Schiller D., Yu A.N.C., Alia-Klein N., Becker S., Cromwell H.C., Dolcos F., Eslinger P.J., Frewen P., Kemp A.H., Pace-Schott E.F. (2024). The Human Affectome. Neurosci. Biobehav. Rev..

[69] Solarz A.K. (1960). Latency of Instrumental Responses as a Function of Compatibility with the Meaning of Eliciting Verbal Signs. J. Exp. Psychol..

[70] Keshava A., Gottschewsky N., Balle S., Nezami F.N., Schüler T., König P. (2023). Action Affordance Affects Proximal and Distal Goal-Oriented Planning. Eur. J. Neurosci..

[71] Van Dessel P., Cummins J., Wiers R.W. (2023). ABC-Training as a New Intervention for Hazardous Alcohol Drinking: Two Proof-of-Principle Randomized Pilot Studies. Addiction.

[72] Markman A.B., Brendl C.M. (2005). Constraining Theories of Embodied Cognition. Psychol. Sci..

[73] Elliot A.J. (1999). Approach and Avoidance Motivation and Achievement Goals. Educ. Psychol..

[74] Elliot A.J., Harackiewicz J.M. (1996). Approach and Avoidance Achievement Goals and Intrinsic Motivation: A Mediational Analysis. J. Pers. Soc. Psychol..

[75] Eder A.B., Elliot A.J., Harmon-Jones E. (2013). Approach and Avoidance Motivation: Issues and Advances. Emot. Rev..

[76] Gibson J.J. (1979). The Ecological Approach to Visual Perception: Classic Edition.

[77] Cesario J., Plaks J.E., Hagiwara N., Navarrete C.D., Higgins E.T. (2010). The Ecology of Automaticity. How Situational Contingencies Shape Action Semantics and Social Behavior: How Situational Contingencies Shape Action Semantics and Social Behavior. Psychol. Sci..

[78] Saraiva A.C., Schüür F., Bestmann S. (2013). Emotional Valence and Contextual Affordances Flexibly Shape Approach-Avoidance Movements. Front. Psychol..

[79] Lee J., Eden A., Park T., Ewoldsen D.R., Bente G. (2022). Embodied Motivation: Spatial and Temporal Aspects of Approach and Avoidance in Virtual Reality. Media Psychol..

[80] Ascheid S., Wessa M., Linke J.O. (2019). Effects of Valence and Arousal on Implicit Approach/Avoidance Tendencies: A fMRI Study. Neuropsychologia.

[81] Zorowitz S., Rockhill A.P., Ellard K.K., Link K.E., Herrington T., Pizzagalli D.A., Widge A.S., Deckersbach T., Dougherty D.D. (2019). The Neural Basis of Approach-Avoidance Conflict: A Model Based Analysis. eNeuro.

[82] Cardinal R.N., Parkinson J.A., Hall J., Everitt B.J. (2002). Emotion and Motivation: The Role of the Amygdala, Ventral Striatum, and Prefrontal Cortex. Neurosci. Biobehav. Rev..

[83] Tye K., Janak P. (2007). Amygdala Neurons Differentially Encode Motivation and Reinforcement. J. Neurosci..

[84] Rolls E.T. (2023). Emotion, Motivation, Decision-Making, the Orbitofrontal Cortex, Anterior Cingulate Cortex, and the Amygdala. Brain Struct. Funct..

[85] Nolte D., Vidal De Palol M., Keshava A., Madrid-Carvajal J., Gert A.L., von Butler E.-M., Kömürlüoğlu P., König P. (2024). Combining EEG and Eye-Tracking in Virtual Reality: Obtaining Fixation-Onset Event-Related Potentials and Event-Related Spectral Perturbations. Atten. Percept. Psychophys..

[86] Garrison K., Schmeichel B., Baldwin C. (2022). Meta-Analysis of the Relationship Between Frontal EEG Asymmetry and Approach/Avoidance Motivation. https://osf.io/preprints/psyarxiv/9tgws.

[87] Vecchio A., Pascalis V. (2020). EEG Resting Asymmetries and Frequency Oscillations in Approach/avoidance Personality Traits: A Systematic Review. Symmetry.

[88] Coan J.A., Allen J.J.B. (2004). Frontal EEG Asymmetry as a Moderator and Mediator of Emotion. Biol. Psychol..

[89] Harmon-Jones E. (2003). Early Career Award. Clarifying the Emotive Functions of Asymmetrical Frontal Cortical Activity. Psychophysiology.

[90] Smith E.E., Reznik S.J., Stewart J.L., Allen J.J.B. (2017). Assessing and Conceptualizing Frontal EEG Asymmetry: An Updated Primer on Recording, Processing, Analyzing, and Interpreting Frontal Alpha Asymmetry. Int. J. Psychophysiol..

[91] Harlé K.M., Bomyea J., Spadoni A.D., Simmons A.N., Taylor C.T. (2020). Proactive Engagement of Cognitive Control Modulates Implicit Approach-Avoidance Bias. Cogn. Affect. Behav. Neurosci..

[92] Elliot A.J., Eder A.B., Harmon-Jones E. (2013). Approach–avoidance Motivation and Emotion: Convergence and Divergence. Emot. Rev..

[93] Jungnickel E., Gehrke L., Klug M., Gramann K., Ayaz H., Dehais F. (2019). Chapter 10—MoBI—Mobile Brain/Body Imaging. Neuroergonomics.

[94] Makeig S., Gramann K., Jung T.-P., Sejnowski T.J., Poizner H. (2009). Linking Brain, Mind and Behavior. Int. J. Psychophysiol..

[95] Gramann K., Ferris D.P., Gwin J., Makeig S. (2014). Imaging Natural Cognition in Action. Int. J. Psychophysiol..

[96] Redcay E., Schilbach L. (2019). Using Second-Person Neuroscience to Elucidate the Mechanisms of Social Interaction. Nat. Rev. Neurosci..

[97] Gramann K., Gwin J.T., Ferris D.P., Oie K., Jung T.-P., Lin C.-T., Liao L.-D., Makeig S. (2011). Cognition in Action: Imaging Brain/body Dynamics in Mobile Humans. Rev. Neurosci..

[98] Hölle D., Meekes J., Bleichner M.G. (2021). Mobile Ear-EEG to Study Auditory Attention in Everyday Life: Auditory Attention in Everyday Life. Behav. Res. Methods.

[99] Maimon G., Straw A.D., Dickinson M.H. (2010). Active Flight Increases the Gain of Visual Motion Processing in Drosophila. Nat. Neurosci..

[100] Kihlstrom J.F. (2021). Ecological Validity and “Ecological Validity”. Perspect. Psychol. Sci..

[101] Grasso-Cladera A., Costa-Cordella S., Rossi A., Fuchs N.F., Parada F.J. (2023). Mobile Brain/Body Imaging: Challenges and Opportunities for the Implementation of Research Programs Based on the 4E Perspective to Cognition. Adapt. Behav..

[102] Stangl M., Maoz S.L., Suthana N. (2023). Mobile Cognition: Imaging the Human Brain in the “real World”. Nat. Rev. Neurosci..

[103] Lang P.J., Bradley M.M., Cuthbert B.N. (1997). International Affective Picture System (IAPS): Technical Manual and Affective Ratings.

[104] Marchewka A., Zurawski Ł., Jednoróg K., Grabowska A. (2014). The Nencki Affective Picture System (NAPS): Introduction to a Novel, Standardized, Wide-Range, High-Quality, Realistic Picture Database. Behav. Res. Methods.

[105] Kim H., Lu X., Costa M., Kandemir B., Adams R.B., Li J., Wang J.Z., Newman M.G. (2018). Development and Validation of Image Stimuli for Emotion Elicitation (ISEE): A Novel Affective Pictorial System with Test-Retest Repeatability. Psychiatry Res..

[106] Grall C., Finn E.S. (2022). Leveraging the Power of Media to Drive Cognition: A Media-Informed Approach to Naturalistic Neuroscience. Soc. Cogn. Affect. Neurosci..

[107] Parada F.J. (2018). Understanding Natural Cognition in Everyday Settings: 3 Pressing Challenges. Front. Hum. Neurosci..

[108] Bradley M.M., Lang P.J. (1994). Measuring Emotion: The Self-Assessment Manikin and the Semantic Differential. J. Behav. Ther. Exp. Psychiatry.

[109] Bynion T.-M., Feldner M.T. (2020). Self-Assessment Manikin. Encyclopedia of Personality and Individual Differences.

[110] Morris J.D. (1995). Observations: SAM: The Self-Assessment Manikin; an Efficient Cross-Cultural Measurement of Emotional Response. J. Advert. Res..

[111] Neamoniti S., Kasapakis V. Hand Tracking vs Motion Controllers: The Effects on Immersive Virtual Reality Game Experience. Proceedings of the 2022 IEEE International Symposium on Multimedia (ISM).

[112] Masurovsky A., Chojecki P., Runde D., Lafci M., Przewozny D., Gaebler M. (2020). Controller-Free Hand Tracking for Grab-and-Place Tasks in Immersive Virtual Reality: Design Elements and Their Empirical Study. Multimodal Technol. Interact..

[113] Roelofs K., Elzinga B.M., Rotteveel M. (2005). The Effects of Stress-Induced Cortisol Responses on Approach-Avoidance Behavior. Psychoneuroendocrinology.

