# Supplementary material for: Approach–Avoidance Bias in Virtual and Real-World Simulations: Insights from a Systematic Review of Experimental Setups
Source: Brain Sci. 2025 Jan 22;15(2):103. doi: 10.3390/brainsci15020103 (PMC11852960; doi:10.3390/brainsci15020103)
Supplement: Supplementary file 1 [file brainsci-15-00103-s001.zip › brainsci-3396319-supplementary/Supplementary Material File S1.pdf]

Supplementary Material File S1

String of search for Web of Science.
((ALL=("approach avoidance bias" OR "approach bias" OR "avoidance bias" OR "motivational bias" OR "approach-avoidance conflict")) OR ALL=("automatic approach bias" OR "automatic bias" OR "approach tendencies" OR "avoidance tendencies")) AND ALL=("virtual reality" OR "VR" OR "immersive environment" OR "virtual environment" OR "augmented reality" OR "mixed reality" OR ‘’natural setup’’ OR ‘’real-world’’).
