# Supplementary material for: Approach–Avoidance Bias in Virtual and Real-World Simulations: Insights from a Systematic Review of Experimental Setups
Source: Brain Sci. 2025 Jan 22;15(2):103. doi: 10.3390/brainsci15020103 (PMC11852960; doi:10.3390/brainsci15020103)
Supplement: Supplementary file 1 [file brainsci-15-00103-s001.zip › brainsci-3396319-supplementary/Supplementary Material File S2.pdf]

Supplementary Material File S2

Data items used to extract information from articles:

1. Aim of the study.
2. Nature of the study.
3. Type of AAT.
4. Stimuli used.
5. Embodied response.
6. Behavioral measure.
7. Comparison.
8. VR system.
9. Resolution.
10. Sampling rate.
11. Controllers used.
12. System to generate the environment.
13. Collection of physiological data (in VR).
14. Participant’s position and movement.
